# Variations in Multiple Syndromic Deafness Genes Mimic Non-syndromic Hearing Loss

**DOI:** 10.1038/srep31622

**Published:** 2016-08-26

**Authors:** G. Bademci, F. B. Cengiz, J. Foster II, D. Duman, L. Sennaroglu, O. Diaz-Horta, T. Atik, T. Kirazli, L. Olgun, H. Alper, I. Menendez, I. Loclar, G. Sennaroglu, S. Tokgoz-Yilmaz, S. Guo, Y. Olgun, N. Mahdieh, M. Bonyadi, N. Bozan, A. Ayral, F. Ozkinay, M. Yildirim-Baylan, S. H. Blanton, M. Tekin

**Affiliations:** 1 John P. Hussman Institute for Human Genomics, University of Miami, Miami, 33136, FL, USA; 2 Division of Genetics, Department of Pediatrics, Ankara University School of Medicine, Ankara, 06620, Turkey; 3 Department of Otolaryngology, Hacettepe University School of Medicine, Ankara, 06100, Turkey; 4 Division of Genetics, Department of Pediatrics, Ege University School of Medicine, Izmir, 35040, Turkey; 5 Department of Otolaryngology, Ege University School of Medicine, Izmir, 35040, Turkey; 6 Department of Otorhinolaryngology, Bozyaka Training and Research Hospital, Bozyaka, Izmir, 35170, Turkey; 7 Department of Radiology, Ege University School of Medicine, Izmir, 35040, Turkey; 8 Koc University School of Medicine, Istanbul, 34450, Turkey; 9 Department of Audiology, Hacettepe University Health Sciences Faculty, Ankara, 06100, Turkey; 10 Rajaie Cardiovascular Medical and Research Center, Iran University of Medical Sciences, Tehran, Iran; 11 Faculty of Natural Sciences, Center of Excellence for Biodiversity, University of Tabriz, Tabriz, Iran; 12 Department of Otolaryngology, Faculty of Medicine, Yuzuncu Yıl University, Van, 65080, Turkey; 13 Department of Otorhinolaryngology, Dicle University School of Medicine, Diyarbakir, 21280, Turkey; 14 Dr. John T. Macdonald Foundation Department of Human Genetics and Department of Otolaryngology, University of Miami, Miami, 33136, FL, USA

## Abstract

The genetics of both syndromic (SHL) and non-syndromic hearing loss (NSHL) is characterized by a high degree of genetic heterogeneity. We analyzed whole exome sequencing data of 102 unrelated probands with apparently NSHL without a causative variant in known NSHL genes. We detected five causative variants in different SHL genes (*SOX10, MITF*, *PTPN11*, *CHD7*, and *KMT2D*) in five (4.9%) probands. Clinical re-evaluation of these probands shows that some of them have subtle syndromic findings, while none of them meets clinical criteria for the diagnosis of the associated syndrome (Waardenburg (*SOX10* and *MITF*), Kallmann (*CHD7* and *SOX10*), Noonan/LEOPARD (*PTPN11*), CHARGE (*CHD7*), or Kabuki (*KMT2D*). This study demonstrates that individuals who are evaluated for NSHL can have pathogenic variants in SHL genes that are not usually considered for etiologic studies.

Hearing loss (HL) affects approximately 1 in 650 children, more than half of which is attributed to genetic factors^1^. Based on distinctive clinical features, approximately 30% of genetic HL is syndromic (SHL) and 70% is non-syndromic (NSHL)^2^. To date, more than 145 NSHL loci have been identified^3^ and HL has been reported as a feature in more than 400 syndromes^4^.

Identifying the specific genetic cause of HL in an individual is difficult due to extensive genetic and clinical heterogeneity. The same pathogenic variant may present differently in either the severity of HL or in the overall clinical presentation^5^,^6^. In this study, we searched for causative DNA variants in a large cohort of individuals who apparently were diagnosed with NSHL after excluding variants in known NSHL genes (Supplementary Table S1).

## Results

**Figure 1:**
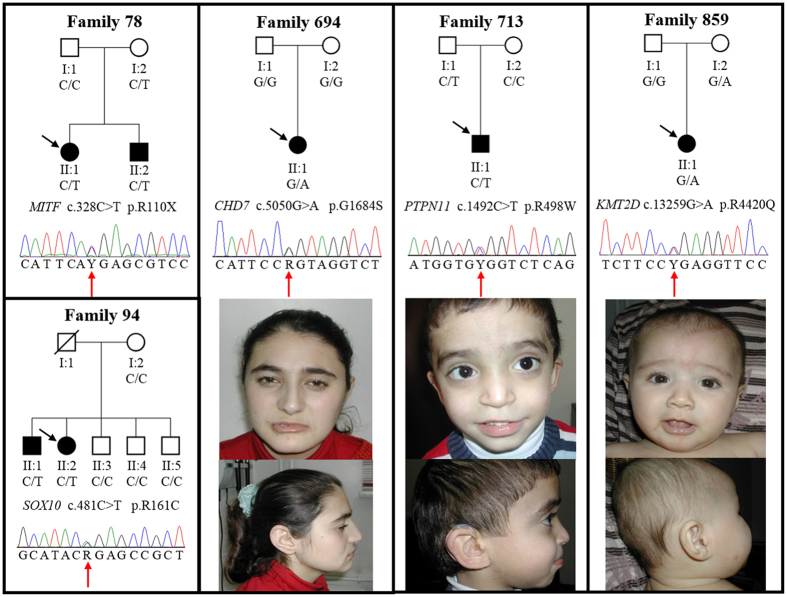
Pedigrees and electropherograms of five families with a causative DNA variant and craniofacial appearance of probands 694, 713, and 859. Black squares and circles denote affected males and females with hearing loss, respectively. Proband 694 has a square face with prominent nasal bridge and columella. Other facial features of CHARGE syndrome such as facial palsy or outer ear findings are missing. Proband 713 has hypertelorism and slightly low-set, posteriorly rotated ears. Other facial features of Noonan/LEOPARD syndrome such as epicanthal folds or ptosis is missing. Proband 859 shows telechantus, epicanthal folds, slightly downslanting palpebral fissures with short columella. Facial features of Kabuki syndrome including long palpebral fissures with eversion of the lateral lower eyelid, arched and broad eyebrows with the lateral third displaying sparseness, and large or prominent ears are missing.

On average, each exome had 99%, 95% and 89% of mappable bases of the Gencode defined exome represented by coverage of 1X, 5X, and 10X reads, respectively. We detected heterozygous pathogenic variants in five different SHL genes that can explain HL in five (4.9%) Turkish families (Table 1). The pathogenic variants *MITF* (MIM 156845) c.328C > T^7^,^8^ (Waardenburg syndrome type II; MIM 193510), *SOX10* (Kallmann syndrome) c.481C > T^9^, *CHD7* (MIM 608892) c.5050G > A^9^,^10^,^11^,^12^ (CHARGE syndrome; MIM 214800 and Kallmann syndrome; MIM 612370), *PTPN11* (MIM 176876) c.1492C > T^13^,^14^,^15^,^16^ (LEOPARD syndrome; MIM 151100), and *KMT2D* (MIM 602113) c.13259G > A^17^ (Kabuki syndrome; MIM 147920) had all been previously reported in patients with syndromic findings. All the identified variants were confirmed with Sanger sequencing in probands and tested for in available family members (Fig. 1).

In family 78, along with the proband, the deaf brother and normal hearing mother both carried the *MITF* c.328C > T variant. Phenotypic features of the 11-year-old female proband and her 7-year-old brother included only congenital severe to profound HL without pigmentary changes in hair, eyes, or skin. The mother was not available for clinical evaluation but denied having pigmentary changes or HL.

In family 94, while both affected children carried the putative pathogenic variant (*SOX10* c.481C > T), the mother did not and the father was unavailable. The mother denied HL in the deceased father. The proband was a 9-year-old female with profound HL. She did not have pigmentary skin, hair, or eye changes. She had a normal neurological examination. The 11-year-old brother had congenital profound deafness with no pigmentary alterations in eyes, skin, or hair.

The proband in family 694 (*CHD7* c.5050G > A variant) had congenital profound deafness associated with inner ear anomalies. At age 18 years her height and weight measured 165 cm and 60 kg, respectively; both were between the 50^th^ and 75^th^ centiles. She was at Tanner 5 stage for puberty and had her menarche at age 14 years. She did not have eye anomalies or a heart defect. She reported normal smelling. She did not have a history of nasal obstruction, nasal discharge, or otitis media. A nasogastric catheter was successfully passed through both nasal openings. While she had mild facial features of CHARGE syndrome (Fig. 1), she did not have other features of CHARGE or Kallmann syndromes (Table 2).

In family 713, the proband and his father carried the *PTPN11* c.1492C > T variant. The proband was a 4-year-old boy with congenital profound deafness. A thorough clinical re-evaluation of the proband detected minor craniofacial features without reaching diagnostic criteria of Noonan or LEOPARD syndromes (Fig. 1, Table 3)^18^,^19^,^20^,^21^,^22^. Father denied having HL and lentigines but was not available for clinical evaluation.

The proband in family 859 had the the *KMT2D* c.13259G > A variant and was a 1-year-old male with profound deafness associated with bilateral cochlear aplasia. The patient was on target for neuromotor development and growth except for speech delay. Further clinical evaluation detected only telecanthus with bilateral epicanthal folds without findings of Kabuki syndrome (Fig. 1). The mother carried the same variant but did not have HL, cognitive problems, or distinctive findings of Kabuki syndrome.

## Discussion

In this study, we detected pathogenic variants in *MITF*, *SOX10, CHD7*, *PTPN11*, and *KMT2D* in patients who were diagnosed as having NSHL. While these genes have been implicated in various forms of SHL, they were not reported to cause NSHL. Re-evaluation of probands shows that some of them exhibit subtle syndromic findings not sufficient to make the clinical diagnosis of the associated syndrome (Supplementary Tables S2 to S5). Importantly, in several families, other individuals carried the same variant but had normal hearing.

Waardenburg syndrome (WS) is the most common type of autosomal dominant SHL with a frequency of 1/40,000 in the general population^23^. WS is primarily characterized by HL and pigmentation anomalies in eyes, skin, and hair^23^,^24^. While there are additional findings affecting face, limbs, or gastrointestinal system in WS types I, III, and IV, WS type II presents only with HL and pigmentary anomalies (Supplementary Table S2). One Chinese and one Caucasian patient with typical pigmentary changes and deafness consistent with WS type II have been reported to be heterozygous for the *MITF* c.328C > T variant^7^,^8^. *SOX10* variants are known to cause WS types II and IV (Hirschsprung disease as an additional finding) (MIM 613266), PCWH (peripheral demyelinating neuropathy, central demyelinating leukodystrophy, Waardenburg syndrome, and Hirschsprung disease) (MIM 609136), and Kallmann syndrome with deafness^25^. It is interesting to note that two patients with *SOX10* mutations resembling NSHL and inner ear anomalies were reported^23^. *SOX10* c.481C > T has been previously reported in a patient with Kallmann syndrome^9^. Our patients with *MITF* and *SOX10* variants did not have any of the pigmentary or neurological findings reported in WS and *SOX10*-related disorders. Formal evaluation of anosmia and hypogonadism reported in these syndromes are difficult to assess in young children and was not attempted.

CHARGE syndrome is autosomal dominant condition with a prevalence of 1 in 10,000 and is characterized by Coloboma of the eye, Heart defects, Atresia of the choanae, Retardation of growth and/or development, Genital and/or urinary tract abnormalities, and Ear anomalies and/or deafness^26^,^27^. Congenital anomalies are usually detected after birth. Overall psychomotor milestones from birth to age of 4 years can be severely delayed, although half of the children with CHARGE syndrome at primary-school age may have satisfactory intellectual ability^28^. Pathogenic variants in *CHD7* cause CHARGE syndrome and hypogonadotropic hypogonadism 5 with anosmia (a.k.a. Kallmann syndrome) or without anosmia (MIM 612370). *CHD7* c.5050G > A has been previously reported to cause CHARGE syndrome (Table 2)^9^,^10^,^11^,^20^. Studies have shown that semicircular canal atresia is a major finding of CHARGE and the patients with semicircular canal atresia should be screened for causative *CHD7* variants^29^.

Noonan syndrome (NS) is an autosomal dominant condition seen 1 in 1,000–2,500 live births. It is characterized by distinctive facial features (low-set, posteriorly rotated ears with fleshy helices, hypertelorism, eyelid ptosis, downslanting palpebral fissures and epicanthal folds), postnatal growth reduction, congenital heart defects, hypertrophic cardiomyopathy, webbing of the neck, and skeletal anomalies^30^. The facial characteristics are usually prominent in early and middle childhood and more subtle in adulthood. Birth weight and length are usually normal. Growth failure is often apparent from the first year of life^31^. Cardiac abnormalities are present in 50–80% of individuals with NS. Median age at diagnosis for hypertrophic cardiomyopathy is five months^32^,^33^. LEOPARD syndrome (LS; multiple Lentigines and café-au-lait spots, Electrocardiographic-conduction abnormalities, Ocular hypertelorism/obstructive cardiomyopathy, Pulmonary stenosis, Abnormalities of the genitalia in males, Retardation of growth, and Deafness) is characterized by multiple lentigines and NS features. Pathogenic variants in *PTPN11* are the most common cause of Noonan and LEOPARD syndromes. The c.1492C > T variant is located in exon 13 which is a hot spot region for this gene. This variant has previously been reported in individuals with LEOPARD syndrome with variable phenotypic features including multiple lentigines, hypertrophic cardiomyopathy, dysmorphic facial features and variable degree of cognitive deficits (Table 3)^13^,^14^,^15^,^16^,^34^.

Kabuki syndrome (KS) is a rare autosomal dominant disorder and is characterized by five cardinal manifestations: a distinctive face (elongated palpebral fissures with eversion of the lateral third of the lower eyelid, arched and broad eyebrows with the lateral third displaying sparseness or notching, short columella with depressed nasal tip, and large, prominent, or cupped ears), skeletal anomalies, digit abnormalities, mild-to-moderate intellectual disability and postnatal growth retardation. While growth is normal at birth, growth failure occurs during the first year of life and becomes more marked with increasing age^20^. Hearing loss is seen in up to 40% of patients, mostly due to recurrent otitis media; sensorineural hearing loss is a rare finding in patients with KS^35^. Pathogenic variants in *KMT2D* and *KDM6A* (MIM 300128) are only the known causes of the disorder^17^. The *KMT2D* c.13259G > A variant has been reported previously in a Korean 3-year-old female with typical facial features including long palpebral fissures with everted lower eyelids, large dysplastic ears, arched eyebrows, flat nasal tip, as well as prominent digital pads, developmental delay and hypotonia without HL^17^ (the variant’s identity was confirmed by the corresponding author via personal communication). Neither parent carried the same variant.

Known examples of genes that can cause both SHL and NSHL include *MYO7A* (MIM 276903), *USH1C* (MIM 605242), *PCDH15* (MIM 605514), *CDH23* (MIM 605516), *CIB2* (MIM 605564), *DFNB31* (MIM 607928) for Usher syndrome (MIM 276900; 276904; 602083; 601067; 614869; 611383), *SLC26A4* (MIM 605646) for Pendred syndrome (MIM 274600), *ACTG1* (MIM 102560) for Baraitser-Winter syndrome 2 (MIM 614583), *TBC1D24* (MIM 613577) for DOOR syndrome (MIM 220500), *KARS* (MIM 601421) for Charcot-Marie-Tooth disease, recessive intermediate B (MIM 613641) and *COL11A2* (MIM 120290) for Stickler syndrome (MIM 184840)^36^,^37^,^38^,^39^,^40^,^41^,^42^,^43^,^44^,^45^,^46^. These genes are usually included in gene panels developed for deafness. *SLC26A4* is one of the most common genes causing autosomal recessive hearing loss with enlarged vestibular aqueduct or Pendred syndrome^5^,^6^,^47^. Even the same pathogenic *SLC26A4* variant can cause nonsyndromic deafness or Pendred syndrome, exemplifying variable phenotypic expressivity of the same gene^48^. Numerous functional studies of *SLC26A4* pathogenic variants suggest that different mechanisms, including endoplasmic reticulum retention and defective plasma membrane targeting of pendrin, residual function of the pendrin, and additional genetic and/or environmental factors might play role in pathogenesis^49^,^50^,^51^.

Our study shows that variants in at least five SHL genes can mimic NSHL. The origin of the variable phenotypes caused by the same variants may be genetic or environmental modifiers. It should be noted that some of the syndromic findings may be age dependent, such as retinitis pigmentosa in Usher syndrome or delayed puberty in Kallmann syndrome, and cannot be detected in a young child with HL. Some children may only have subtle phenotypic features of a syndrome, such as mild facial features of CHARGE or Noonan syndrome without meeting diagnostic criteria. Clinical investigation for every potential finding of over 400 syndromes is not practical during the etiological evaluation of an individual with HL. Moreover, laboratory evaluations would yield equivocal results as recently reported for Jervell and Lange-Nielsen syndrome (MIM 220400) in which interpretation of EKG findings was complicated by sinus arrhythmia^52^. We recommend that consideration be given to inclusion of all genes known to cause any form of HL in gene panels developed for hereditary deafness. Alternatively all-inclusive genome analysis such as whole exome or whole genome sequencing should be considered when deafness gene panel studies prove inconclusive.

## Methods

### Statement of Ethics

This study was approved by the University of Miami Institutional Review Board (USA), the Growth and Development Research Ethics Committee (Iran), and Ankara University Medical School Ethics Committee (Turkey). Methods were carried out in accordance with the approved guidelines. A signed informed consent form was obtained from each participant or, in the case of a minor, from parents.

### Subjects

This study includes 102 probands selected from a larger cohort that has been found to be negative for all known NSHL genes initially by Sanger sequencing (for variants in *GJB2* and mtDNA 1555A > G) and subsequently via whole exome sequencing (for a list of screened genes see Supplementary Table S1). Families were from Turkey (77), Iran (18) and the USA (7). Probands were the only affected individuals in 54 families (simplex); there were at least 2 affected siblings born to unaffected parents in the remaining 48 families (multiplex) suggesting autosomal recessive inheritance. In all cases, HL was congenital or prelingual in onset with a severity ranging from severe to profound. Clinical evaluations of all probands were performed by both an otorhinolaryngologist and a clinical geneticist. These evaluations included a thorough physical examination, otoscopy, and ophthalmoscopy. An EKG, urinalysis, and, when available, high resolution CT of the temporal bone or MRI were performed in probands. Blood was collected from probands and family members (parents and siblings) when available and DNA was extracted from peripheral leukocytes by standard protocols.

### Whole-Exome Sequencing and Bioinformatic Analysis

Agilent SureSelect Human All Exon 50 Mb and a HiSeq 2000 instrument (Illumina) were used for capture and sequencing based on our previously published protocol^47^. Variants were called using FreeBayes^53^. After overall quality checks, Genesis 2.0 (https://www.genesis-app.com/) was used for variant filtering. We filtered variants in all morbid OMIM genes (except for the previously screened known NSHL genes -Supplemental Table S1) with minor allele frequency thresholds of 0.005 for recessive and 0.0005 for dominant variants as recommended^54^. For allele frequencies we used ExAC (http://exac.broadinstitute.org/) and EVS (http://evs.gs.washington.edu/EVS/) databases as well as our internal whole exome sequencing database that includes more than 300 individuals for each studied population. SIFT (http://sift.jcvi.org/), Polyphen2 (http://genetics.bwh.harvard.edu/pph2/) and MutationTaster (http://www.mutationtaster.org/) were used for *in silico* analysis. In addition, XHMM and Conifer were used for the Copy Number Variant (CNVs) detection^55^,^56^,^57^. Only genes that have been reported to have HL as a phenotypic finding in OMIM were further selected. Variants meeting these criteria were further annotated based on their presence and pathogenicity information in Human Gene Mutation Database (HGMD) and ClinVar^58^,^59^. In addition American College of Medical Genetics and Genomics (ACMG) Standards and Guidelines 2015 were used for the variant interpretation^60^. Probands who were found to carry variants in SHL genes were subsequently re-evaluated for syndromic findings.

## Additional Information

**How to cite this article**: Bademci, G. *et al*. Variations in Multiple Syndromic Deafness Genes Mimic Non-syndromic Hearing Loss. *Sci. Rep*. **6**, 31622; doi: 10.1038/srep31622 (2016).

## Supplementary Material

Supplementary Information

## Figures and Tables

**Table 1:** Causative variants identified in this study.

Family ID	Gene	Transcript ID	cDNA	Protein	ClinVar	HGMD	ExAC	PolyPhen2	SIFT	Mutation Taster	ACMG
78	*MITF*	NM_000248.3	c.328C > T	p.R110X	n.a.	P	n.a.	n.a.	n.a.	DC	P
94	*SOX10*	NM_006941.3	c.481C > T	p.R161C	n.a.	P	n.a.	probably-damaging	DM	DC	LP
694	*CHD7*	NM_017780.3	c.5050G > A	p.G1684S	n.a.	P	n.a.	probably-damaging	DM	DC	P
713	*PTPN11*	NM_002834.3	c.1492C > T	p.R498W	P	P	n.a.	probably-damaging	DM	DC	P
859	*KMT2D*	NM_003482.3	c.13259G > A	p.R4420Q	n.a.	P	2.54E-05	possibly-damaging	DM	DC	LP

n.a.: not available, P: Pathogenic, LP: Likely Pathogenic, DC: Disease Causing, DM: Damaging, ExAC: Exome Aggregation Consortium, HGMD: Human Gene Mutation Database, ACMG: American College of Medical Genetics (Richards *et al*.)^60^.

**Table 2:** Phenotype of patients with the *CHD7* c.5050G > A variant based on Blake’s (Blake *et al*.)^18^ CHARGE criteria*.

	Asakura *et al*.,^10^ Fujita *et al*.^12^	Husu *et al.^11^*	Marcos *et al*.^9^	Proband 694
*Major Criteria*	8.5 yo, F	8 yo, F	39 yo, M	18 yo, F
1. Coloboma, micropthalmia	−	−	−	−
2. Choanal atresia or stenosis	−	−	−	−
3. Characteristic external ear anomaly, middle/inner ear malformations, mixed deafness	+(abnormal semicircular canals)	+(hypoplastic/absent semicircular canals)	n.a.	+(bilateral cochlear incomplete partition type I, semicircular canal dysplasia)
4. Cranial nerve dysfunction	+(aplasia of olfactory bulb)	+(vestibular dysfunction, swallowing problems, sensorineural HL)	+(anosmia, HL)	+(sensorineural HL)
*Minor Criteria*				
1. Cardiovascular malformations	+	+	+	−
2. Tracheo-oesophageal defects	n.a.	n.a.	n.a.	−
3. Genital hypoplasia or delayed pubertal development	−	n.a.	+(genital hypoplasia)	−
4. Cleft lip and/or palate	+	+(unilateral cleft lip and palate)	−	−
5. Developmental delay	+	+	+	+(speech and mild gross motor)
6. Growth retardation	+	+	n.a.	−
7. Characteristic face	+	+	n.a.	+(mild facial features)

n.a.: not available, yo: years old, HL: Hearing loss.

^*^For diagnosis of typical CHARGE 4 major or 3 major + 3 minor criteria are needed. Probable/possible CHARGE syndrome: 1 or 2 major and several minor criteria.

**Table 3:** Phenotypic features reported in individuals carrying *PTPN11* p.R498W variant.

	Sarkozy *et al*.^16^	Sarkozy *et al*.^16^	Digilio *et al*.^34^	Kratz *et al*.^15^	Edwards *et al*.^14^	Proband 713
Cardinal Features of Noonan/LEOPARD syndrome	2 yo, F	34 yo, F	<1 yo, n.a.	1 < yo, M	5 yo, M	4 yo, M
Multiple Lentigines	−	−	+	n.a.	−	−
Cardiac abnormalities, particularly hypertrophic cardiomyopathy	+(h.c.m.)	+(h.c.m.)	+(h.c.m.)	+(h.c.m.)	+(pulmonic valve stenosis)	−
Poor linear growth/short stature	n.a.	n.a.	+	n.a.	+	− (height is at the 50^th^ centile)
Pectus deformity	n.a.	n.a.	n.a.	−	+(mild pectus deformity)	−
Dysmorphic facial features, including widely spaced eyes and ptosis	+	+	n.a.	−	+(mild dysmorphic facial features)	mild dysmorphic facial features (hypertelorism and low set posteriorly rotated ears)
Additional features occurring frequently in LEOPARD syndrome						
Variable degree of cognitive deficits	+	+	n.a.	n.a.	+(mild global developmental delay)	−
Sensorineural hearing loss	−	−	−	−	−	+
Cryptorchidism	n.a.*	n.a.*	n.a.	n.a.	+	−
Skeletal anomalies	n.a.	n.a.	n.a.	−	n.a.	−
Café-au-lait macules	+	−	+	n.a.	−	−
Other Information	−	Patient 1’s mother	−	Died at 2 months with juvenile myelomonocytic leukemia	−	−

n.a.: not available, n.a.*: not applicable, h.c.m.: hypertrophic cardiomyopathy, yo: years old, M: Male, F: Female.
