# Supplementary material for: Variations in Multiple Syndromic Deafness Genes Mimic Non-syndromic Hearing Loss
Source: Sci Rep. 2016 Aug 26;6:31622. doi: 10.1038/srep31622 (PMC4999867; doi:10.1038/srep31622)
Supplement: Supplementary Information [file srep31622-s1.pdf]

SUPPLEMENTARY INFORMATION

Variations in Multiple Syndromic Deafness Genes Mimic Non-syndromic Hearing Loss

Guney Bademci[1], Filiz B. Cengiz[1], Joseph Foster II[1], Duygu Duman[2], Levent Sennaroglu[3], Oscar Diaz-Horta[1], Tahir Atik[4], Tayfun Kirazli[5], Levent Olgun[6], Hudaver Alper[7], Ibis Menendez[1], Ilayda Loclar[8], Gonca Sennaroglu[9], Suna Tokgoz-Yilmaz[9], Shengru Guo[1], Yuksel Olgun[6], Nejat Mahdieh[10], Mortaza Bonyadi[11], Nazim Bozan[12], Abdurrahman Ayral[12], Ferda Ozkinay[4], Muzeyyen Yildirim-Baylan[13], Susan H. Blanton[1,14], Mustafa Tekin[1,14]*

1) John P. Hussman Institute for Human Genomics, University of Miami, Miami, 33136, FL, USA,

2) Division of Genetics, Department of Pediatrics, Ankara University School of Medicine, Ankara, 06620, Turkey

3) Department of Otolaryngology, Hacettepe University School of Medicine, Ankara, 06100, Turkey

4) Division of Genetics, Department of Pediatrics, Ege University School of Medicine, Izmir, 35100, Turkey

5) Department of Otolaryngology, Ege University School of Medicine, Izmir, 35100, Turkey

6) Department of Otorhinolaryngology, Bozyaka Training and Research Hospital, Bozyaka, Izmir, 35170, Turkey

7) Department of Radiology, Ege University School of Medicine, Izmir, 35100, Turkey

8) Koc University School of Medicine, Istanbul, 34450, Turkey

9) Department of Audiology, Hacettepe University Health Sciences Faculty, Ankara, 06100, Turkey

10) Rajaie Cardiovascular Medical and Research Center, Iran University of Medical Sciences, Tehran, Iran

11) Faculty of Natural Sciences, Center of Excellence for Biodiversity, University of Tabriz, Tabriz, Iran

12) Department of Otolaryngology, Faculty of Medicine, Yuzuncu Yıl University, Van, 65080, Turkey

13) Department of Otorhinolaryngology, Dicle University School of Medicine, Diyarbakir, 21280, Turkey

14) Dr. John T. Macdonald Foundation Department of Human Genetics and Department of Otolaryngology, University of Miami, Miami, 33136, FL, USA

Corresponding author: Mustafa Tekin, M.D, 1501 NW 10th Avenue, BRB-610 (M-860)**,** Miami, FL, 33136 Phone: 305-243-2381**,** E-mail: mtekin@miami.edu

Table S1: List of the NSHL genes prescreened via whole exome sequencing*

	**Gene (MIM ID)**
**Autosomal Recessive NSHL genes**	*ADCY1* (MIM 103072), *BDP1* (MIM 607012), *CABP2* (MIM 607314), *CDH23* (MIM 605516), *CIB2* (MIM 605564), *CLDN14* (605608), *CLIC5* (MIM 607293), *COL11A2* (MIM 120290), *ELMOD3* (MIM 615427), *EPS8* (MIM 600206), *ESPN* (MIM 606351), *ESRRB* (MIM 602167), *FAM65B* (MIM 611410), *GIPC3* (MIM 608792), *GJB2* (MIM 121011), *GJB3* (MIM 603324), *GJB6* (MIM 604418), *GPR98* (MIM 602851), *GRXCR1* (MIM 613283), *GRXCR2* (MIM 615762), *HGF* (MIM 142409), *ILDR1* (MIM 609739), *KARS* (MIM 601421), *LHFPL5* (MIM 609427), *LOXHD1* (MIM 613072), *LRTOMT* (MIM 612414), *MARVELD2* (MIM 610572), *MSRB3* (MIM 613719), *MYO15A* (MIM 602666), *MYO3A* (MIM 606808), *MYO6* (MIM 600970), *MYO7A* (MIM 276903), *OSBPL2* (MIM 606731), *OTOA* (MIM 607038), *OTOF* (MIM 603681), *OTOG* (MIM 604487), *OTOGL* (MIM 614925), *PCDH15* (MIM 605514), *PJVK* (MIM 610219), *PNPT1* (MIM 610316), *PTPRQ* (MIM 603317), *RDX* (MIM 179410), *SERPINB6* (MIM 173321), *SLC26A4* (MIM 605646), *SLC26A5* (MIM 604943), *STRC* (MIM 606440), *SYNE4* (MIM 615535), *TBC1D24* (MIM 613577), *TECTA* (MIM 602574), *TMC1* (MIM 606706), *TMIE* (MIM 607237), *TMPRSS3* (MIM 605511), *TPRN* (MIM 613354), *TRIOBP* (MIM 609761), *TSPEAR* (MIM 612920), *USH1C* (MIM 605242), *USH1G* (MIM 607696) and *WHRN* (MIM 607928)
**Autosomal Dominant NSHL genes**	*ACTG1* (MIM 102560), *CCDC50* (MIM 611051), *CEACAM16* (MIM 614591), *COCH* (MIM 603196), *COL11A2* (MIM 120290), *CRYM* (MIM 123740), *DFNA5* (MIM 608798), *DIAPH1* (MIM 602121), *EYA4 (MIM 603550), GJB2* (MIM 121011), *GJB3* (MIM 603324), *GJB6* (MIM 604418), *GRHL2* (MIM 608576), *HOMER2* (MIM 604799), *KCNQ4* (MIM 603537), *MYH14* (MIM 608568), *MYH9* (MIM 160775), *MYO6* (MIM 600970), *MYO7A (MIM 276903), OSBPL2* (MIM 606731), *P2RX2* (MIM 600844), *POU4F3* (MIM 602460), *SIX1* (MIM 601205), *SLC17A8* (MIM 607557), *SMAC/DIABLO* (MIM 605219), *TBC1D24* (MIM 613577), *TECTA* (MIM 602574), *TJP2* (MIM 607709), *TMC1* (MIM 606706), *TNC* (MIM 187380) and *WFS1* (MIM 606201)
**X-Linked NSHL genes**	*COL4A6* (MIM 303631), *POU3F4* (MIM 300039), *PRPS1* (MIM 311850) and *SMPX* (MIM 300226)

*The gene list is created by using www.hereditaryhearingloss.org and OMIM (http://omim.org/)

Table S2. Diagnostic Criteria for Waardenburg Syndrome Type II*

**Criteria WS Type II**	**Proband 78**	**Proband 94**
**1.** Congenital sensorineural hearing loss	+	+
**2.** Pigmentary disturbances of iris	-	-
**a.** Complete heterochromia iridum-two eyes of different color	-	-
**b.** Partial or segmental heterochromia- segments of blue or brown pigmentation in one or both eyes	-	-
**c.** Hypoplastic blue eyes-characteristic brilliant blue in both eyes	-	-
**3.** Pigmentary disturbances of the hair	-	-
**a.** White forelock from birth or in teens	-	-
**b.** Premature greying before age 30 years	-	-
**4.** A first or second degree relative with two or more of criteria 1-3	-	-

*A person should fulfill at least two of the criteria to be counted as affected.

Table S2 Reference: Liu, X.Z., Newton, V.E. & Read, A.P. Waardenburg syndrome type II: phenotypic findings and diagnostic criteria. Am J Med Genet **55**, 95-100 (1995).

Table S3. CHARGE Syndrome Diagnostic Criteria (Verloes Criteria)*

	**Proband 694**
**Major signs ("the 3 C")**	
Coloboma (iris or choroid, with or without microphthalmia)	-
Atresia of Choanae	-
Hypoplastic semi-circular Canals	+
**Minor signs**	
Rhombencephalic dysfunction (brainstem dysfunctions, cranial nerve VII to XII palsies and neurosensory deafness)	+ (neurosensory deafness)
Hypothalamo-hypophyseal dysfunction (including GH and gonadotrophin deficiencies)	-
Abnormal middle or external ear	-
Malformation of mediastinal organs (heart, esophagus)	-
Mental retardation	-

*Inclusion rule;

Typical CHARGE: 3 majors signs or 2/3 major signs + 2/5 minor signs

Partial/incomplete CHARGE: 2/3 major + 1/5 minor

Atypical CHARGE: 2/3 major + 0/5 minor or 1/3 major + 3/5 minors

Table S3 Reference: Verloes, A. Updated diagnostic criteria for CHARGE syndrome: a proposal. Am J Med Genet A **133A**, 306-8 (2005).

Table S4. Diagnostic criteria for Noonan syndrome (NS)

Feature	A = Major	B = Minor	Proband 713
**1** Facial	Typical face dysmorphology	Suggestive face dysmorphology	1B
**2** Cardiac	Pulmonary valve stenosis, HOCM and/or ECG typical of NS	Other defect	-
**3** Height	<P3*	<P10*	-
**4** Chest wall	Pectus carinatum/excavatum	Broad thorax	-
**5** Family history	First degree relative with definite NS	First degree relative with suggestive NS	-
**6** Other	Mental retardation, cryptorchidism and lymphatic dysplasia	One of mental retardation, cryptorchidism, lymphatic dysplasia	-

HOCM: hypertrophic obstructive cardiomyopathy;

*P3 and P10 refer to percentile lines for height according to age, with the normal range of variation defined as P3-P97 inclusive

Definitive NS: 1 "A" plus one other major sign or two minor signs; 1 "B" plus two major signs or three minor signs

Table S4 Reference: van der Burgt, I. Noonan syndrome. Orphanet J Rare Dis **2**, 4 (2007).

Table S5. Diagnostic Criteria of the Kabuki Syndrome (five cardinal symptoms of Kabuki syndrome)

**Typical facial features:**	**Proband 859**
Elongated palpebral fissures with eversion of the lateral third of the lower eyelid	-
Arched and broad eyebrows with the lateral third displaying sparseness or notching	-
Short columella with depressed nasal tip	-
Large, prominent, or cupped ears	-
**Skeletal anomalies:**	
Spinal column abnormalities, including sagittal cleft vertebrae, butterfly vertebrae, narrow intervertebral disc space, and/or scoliosis	-
Brachydactyly V	-
Brachymesophalangy	-
Clinodactyly of fifth digits	-
**Dermatoglyphic abnormalities:** persistence of fetal fingertip pads	-
Note: While absence of digital triradius c and/or d and increased digital loop and hypothenar loop patterns can be observed, this type of analysis is not routinely done in clinical practice in most centers.	-
**Mild to moderate intellectual disability**	-
**Postnatal growth deficiency**	-

Table S5 Reference: Niikawa, N. et al. Kabuki make-up (Niikawa-Kuroki) syndrome: a study of 62 patients. Am J Med Genet **31**, 565-89 (1988).
